# Discovery of proangiogenic CD44+mesenchymal cancer stem cells in an acute myeloid leukemia patient’s bone marrow

**DOI:** 10.1186/s13045-020-00899-x

**Published:** 2020-06-03

**Authors:** Huynh Cao, Jeffrey Xiao, Mark E. Reeves, Kimberly Payne, Chien Shing Chen, David J. Baylink, Guido Marcucci, Yi Xu

**Affiliations:** 1 Division of Hematology and Oncology, Loma Linda University Medical Center, Loma Linda, CA USA; 2 Regenerative Medicine, Department of Medicine, Loma Linda University, Loma Linda, CA USA; 3 Division of Anatomy, Department of Basic Sciences, Loma Linda University, Loma Linda, CA USA; 4 Gehr Family Center for Leukemia Research, Hematology Malignancies and Stem Cell Transplantation Institute, City of Hope Medical Center, Duarte, CA USA

**Keywords:** Acute myeloid leukemia, Mesenchymal stem cells, Mesenchymal cancer stem cells, CD44, Angiogenesis, Microenvironment

## Abstract

Here, we report a unique acute myeloid leukemia (AML) bone marrow-derived mesenchymal stem cell (MSC) with both mesenchymal and endothelial potential, which we have named Mesenchymal Cancer Stem Cells (MCSCs). These MCSCs are CD90-CD13-CD44+ and differ from MSCs in isolation, expansion, differentiation, immunophenotype, and cytokine release profile. Furthermore, blocking CD44 inhibited the proliferation and cluster formation of early MCSCs with lower ICAM-1 protein levels. Similar CD90-CD44+ cancer stem cells have been reported in both gastric and breast cancers, which grew in floating spheres in vitro and exhibited mesenchymal features and high metastatic/tumorigenic capabilities in vivo. Our novel discovery provides the first evidence that certain AMLs may be comprised of both hematopoietic and stromal malignant cells. Targeting MCSCs and their cytokine release has potential as a novel therapeutic approach in AML.

To the Editor

Acute myeloid leukemia (AML) most commonly occurs in older adults [1] and has the lowest survival rate among all types of leukemia [2]. Mechanistically, AML is a hematopoietic cancer that involves a complex interplay among different types of bone marrow (BM) cells, cytokines, growth factors, and neural modulation—all of which contribute to the survival and growth of malignant Hematopoietic Stem Cells (HSCs) [3, 4]. Currently, most AML therapies have been developed to target malignant HSCs because of their significant role in initiating uncontrolled clonal proliferation and transforming the naïve BM niche to allow disease progression and treatment resistance [5]. However, the overall survival of relapsed or refractory AML patients has remained dismal for the past three decades [6]. Therefore, more research on the mechanisms of AML etiology and relapse are urgently needed to further the development of effective treatments.

A new concept of niche-induced oncogenesis has been proposed that is based on the observation that artificial genetic perturbation of mesenchymal stem cells (MSCs), and their progenies leads to leukemogenesis of myelodysplasia and AML in transgenic mice [7, 8]. In this study from an AML patient BM, for the first time, we discovered previously unrecognized CD90-CD13-CD44+ MSCs with tumorigenic proliferation and proangiogenic properties ex vivo.

The AML bone marrow donor is a 32-year-old female with a past medical history of diabetes and hypothyroidism. She was admitted to the emergency department with intermittent chest pain, shortness of breath, and gum bleeding, and diagnosed with AML with myelomonocytic differentiation. Peripheral blood smears showed leukocytosis with ~ 52% blasts/equivalents and marked thrombocytopenia. FISH showed normal cytogenetics. An AML panel was positive for mutations of DNMT3A, NPM1, and NRAS without FLT3-ITD.

During ex vivo isolation of MSCs from this patient BM, we observed two different cell populations. The early plastic-attached cells were CD90+CD13+CD44+ MSCs (Fig. 1(a1, a3, d1 upper panels)). The late attached cells in the non-adherent floating cells of MSC cultures became adherent after 7 or more days (Fig. 1(a2)). We named them mesenchymal cancer stem cells (MCSCs) because of their capabilities of multiple lineage differentiation (Supplementary Figure 1), rapid cell proliferation and self-renewal (Fig. 1b, c, Supplementary Figure 2), and release of high amounts of cytokines and growth factors reported to be essential for angiogenesis and blasts’ growth. At early passages (P), there were about 20% CD90-CD13-CD44+ cells within large-sized strong CD44+ MCSCs (Fig. 1d lower panels). MCSCs grew in plastic attachment patterns; however, the number of viable floating cells increased with passage (Fig. 1(A4)). After P7, most MCSCs were CD90-CD13-CD44+, expanded in floating cluster conditions (Fig. 1g, i), and generated tube-like vessel structures (Fig. 1j) by expressing endothelial cell biomarkers like VE-Cadherin/CD144 (Fig. 1k). To understand the interaction between MCSCs and AML blasts, we plated MCSCs or MSCs with GFP-MOLM-14 (AML blasts) together. After 24 h of 1:1 co-cultures, GFP-MOLM-14 were found in the middle of floating clusters and surrounded by MCSCs (Fig. 1m), in contrast to their position on top of MSCs when cultured with MSCs only (Fig. 1l). These data are consistent with a previous report that AML blasts integrate into vascular niches to progress and metastasize [9].

**Fig. 1:**
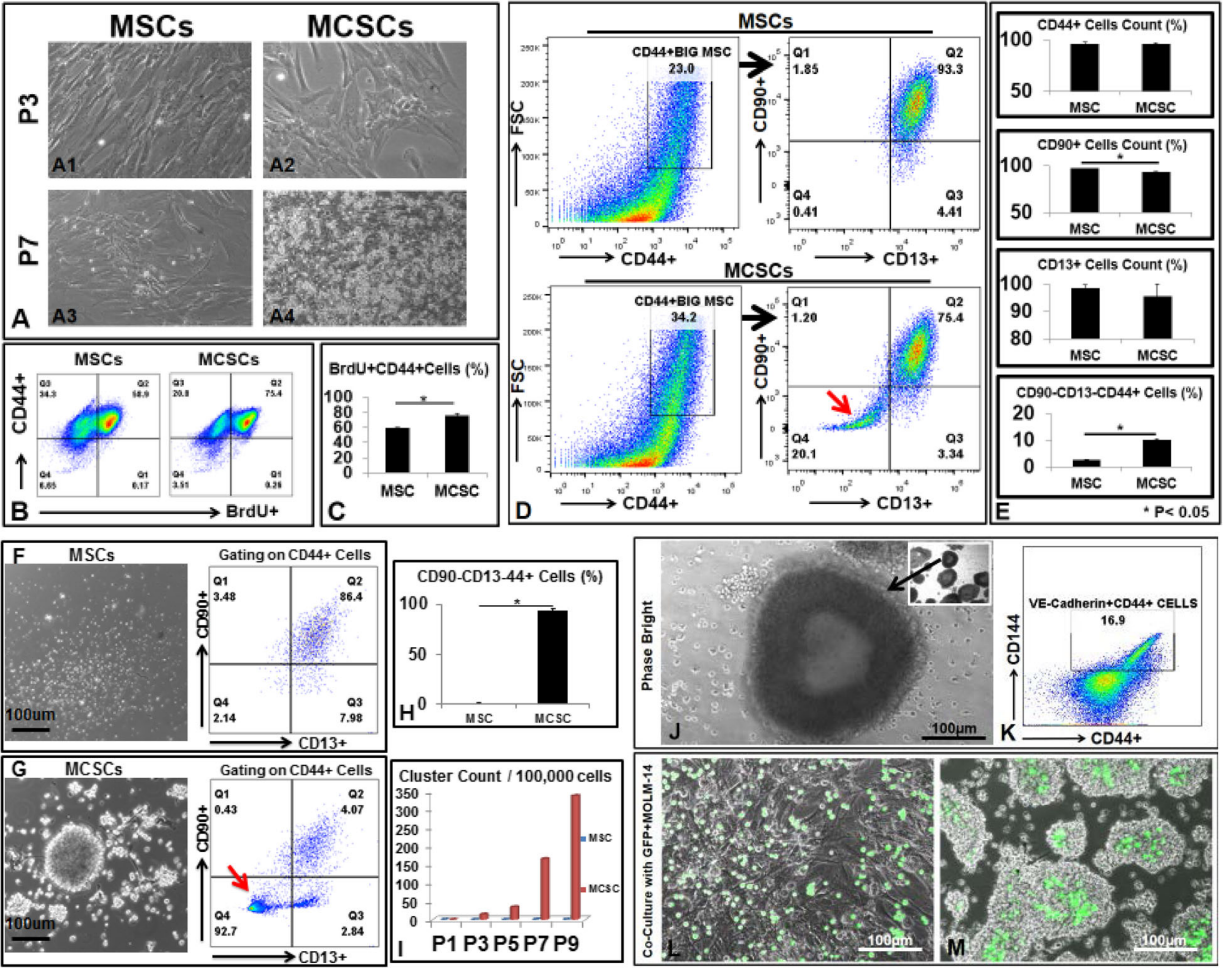
Isolation and expansion of CD90-CD13-CD44+MCSCs from an AML patient bone marrow ex vivo. **a** Phase bright images of MSCs and MCSCs at different passages (P). **b** 24 h after culturing cells with BrdU, P7 MSCs, and P7 MCSCs were collected and analyzed by FACS for incorporation of BrdU. **c** Aggregate data showing percentage of BrdU+CD44+ cells in MSCs and MCSCs. **d** P3 MSCs and P3 MCSCs were analyzed by FACS for immunophenotype. Thick black arrows indicate gating strategy. The red arrow indicates the population of CD90-CD13-CD44+ cells. **e** Aggregate FACS data showing the percentage of MSCs and MCSCs positive for each biomarker. **f** Left: Phase bright image of floating cells from P5 MSC culture. Right: P5 MSCs were collected and analyzed by FACS. **g** Left: Phase bright image of floating cells from P5 MCSC culture. Right: P5 MCSCs were collected and analyzed by FACS. The red arrow indicates CD90-CD13-CD44+ cells. **h***A*ggregate FACS data showing the percentage of CD90-CD13-CD44+ cells comprising the floating cell populations from MSCs and MCSCs. **i***C*umulative counts of clusters generated from MSC and MCSC cultures over time. **j** Inset: phase bright images of floating tube-like structure from P8 MCSC culture. Black thick arrows indicate the same round tube at × 2 and × 10 magnification. **k** P8 Floating MCSCs were analyzed by FACS for endothelial cell markers such as VE-Cadherin (CD144). **l** Phase bright image of GFP+MOLM-14 cells cultured with MSCs after 24-h plating. **m** Phase bright image of GFP+MOLM-14 cells co-cultured with MCSCs after 24-h plating. Where applicable, data are means ± SEM from each group and were analyzed by Student *t* test. *p < 0.05; N=3. Scale bar 100 μm

Finally, we performed a proteome assay of supernatants from MSC and MCSC cultures. The release of key angiogenic cytokines like VEGF and growth factors such as FGF basic and PDGF were significantly increased in the P7 MCSC culture (Fig. 2b). To evaluate therapeutic potential, we performed blocking experiments with anti-CD44 monoclonal antibodies, which stopped tumorigenic proliferation of P5 MCSCs, but not P7 MCSCs (Supplementary Figure 3). It is possible that the large amount of ICAM-1 (53-fold increase in P7 MCSCs versus P5 MCSCs, Supplementary Figure 3E) compensated the loss of CD44 as previously reported in CD44 null mice [10].

**Fig. 2:**
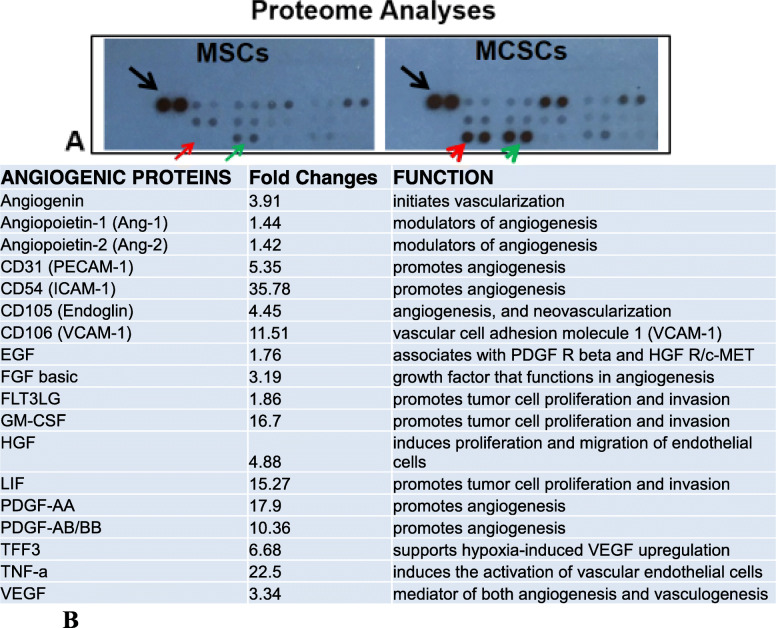
Proteome analyses indicate the significant increase in angiogenic protein release from MCSC cultures. **a** Image of partial blot films developed for proteome analyses. The black arrow indicates the control dots from the manufacturer. The red arrow indicates no protein expression. The red arrowhead indicates protein expression at the same location in the film. The green arrow indicates weak protein expression. The green arrowhead indicates strong protein expression at the same location. Note: each antibody has two dot spots according to manufacturer’s specification. **b** Proteome comparison (fold change) of angiogenic proteins between supernatants from P7 MSC and P7 MCSC cultures. Fold Changes represent MCSCs versus MSCs

In summary, we provide novel evidence of MCSCs as the cellular origin of angiogenesis and cytokine release in an AML patient’s BM. More research with additional patients is needed to discover if this phenomenon is unique to this one AML patient or has broader ramifications for AML as a whole. Our data suggest that future therapeutic strategies should also be developed to target MCSCs and their cytokine release to achieve stringent complete remission and prevent AML progression and relapse. The existence of MCSCs might be applicable to other cancers as well.

## Supplementary information

**Additional file 1: Supplementary Figures. Supplementary Figure 1. ** There is no significant difference in differentiation capabilities of P4 MSCs and P4 MCSCs during *ex vivo* cultures. *A)* MSCs Panel: FACS plot of CD34-CD13+MSCs, which differentiated into bone (Alizalin staining), fat (phase bright), and cartilage (Alcian blue staining). *B)* MCSCs Panel: FACS plot of CD34-CD13+MCSCs, which differentiated into bone (Alizalin staining), fat (phase bright), and cartilage (Alcian blue staining). **Supplementary Figure 2.** Comparison of Cell Proliferation of MSCs and MCSCs. *A)* The P10 MCSCs were found to proliferate much faster than P10 MSCs. ** P<0.01; *B)* These rapidly proliferating MCSCs do not express cleaved Caspase3 (Cell Signaling Technology, Cat#9664S) and continue to express strong CD44+. **Supplementary Figure 3.** Anti-CD44 monoclonal antibodies inhibited the cluster formation and proliferation of P5 MCSCs. *A)* Phase bright images of floating clusters from P5 MCSCs without treatment. *B)* Phase bright images of floating cells from P5 MCSCs with treatment of anti-CD44. *C)* Aggregate cluster count data from P5 MCSCs treated with anti-CD44 or without treatment. *D)* Aggregate cluster count data from P7 MCSCs treated with anti-CD44 or without treatment. Where applicable, data are means ± SEM from each group and were analyzed by Student t-test. *P<0.05; N=3. *E)* Proteome comparison (mean pixel density) of ICAM-1 between supernatants from P7 MCSC and P5 MCSC cultures. *P<0.05

**Additional file 2: Materials and Methods.** Discovery of proangiogenic CD44+mesenchymal cancer stem cells in an acute myeloid leukemia patient’s bone marrow

## Data Availability

The datasets used and/or analyzed in the current study are available from the corresponding author.

## Abbreviations

AML: Acute myeloid leukemia
MSC: Mesenchymal stem cell
MCSCs: Mesenchymal cancer stem cells
BM: Bone marrow
HSCs: Hematopoietic stem cells
FACS: Flow cytometry
P: Passages

## Supplementary information

**Supplementary information** including Materials and Methods (Additional file 2) accompanies this paper at 10.1186/s13045-020-00899-x.
