# Supplementary material for: Discovery of proangiogenic CD44+mesenchymal cancer stem cells in an acute myeloid leukemia patient’s bone marrow
Source: J Hematol Oncol. 2020 Jun 3;13:63. doi: 10.1186/s13045-020-00899-x (PMC7268388; doi:10.1186/s13045-020-00899-x)
Supplement: Supplementary file 1 — Additional file 1: Supplementary Figures. Supplementary Figure 1. There is no significant difference in differentiation capabilities of P4 MSCs and P4 MCSCs during ex vivo cultures. A) MSCs Panel: FACS plot of CD34-CD13+MSCs, which differentiated into bone (Alizalin staining), fat (phase bright), and cartilage (Alcian blue staining). B) MCSCs Panel: FACS plot of CD34-CD13+MCSCs, which differentiated into bone (Alizalin staining), fat (phase bright), and cartilage (Alcian blue staining). Supplementary Figure 2. Comparison of Cell Proliferation of MSCs and MCSCs. A) The P10 MCSCs were found to proliferate much faster than P10 MSCs. ** P<0.01; B) These rapidly proliferating MCSCs do not express cleaved Caspase3 (Cell Signaling Technology, Cat#9664S) and continue to express strong CD44+. Supplementary Figure 3. Anti-CD44 monoclonal antibodies inhibited the cluster formation and proliferation of P5 MCSCs. A) Phase bright images of floating clusters from P5 MCSCs without treatment. B) Phase bright images of floating cells from P5 MCSCs with treatment of anti-CD44. C) Aggregate cluster count data from P5 MCSCs treated with anti-CD44 or without treatment. D) Aggregate cluster count data from P7 MCSCs treated with anti-CD44 or without treatment. Where applicable, data are means ± SEM from each group and were analyzed by Student t-test. *P<0.05; N=3. E) Proteome comparison (mean pixel density) of ICAM-1 between supernatants from P7 MCSC and P5 MCSC cultures. *P<0.05 [file 13045_2020_899_MOESM1_ESM.pdf]

Discovery of Proangiogenic CD44+Mesenchymal Cancer Stem Cells in an Acute Myeloid Leukemia Patient's Bone Marrow

Authors:

Huynh Cao[1*], Jeffrey Xiao[2*], Mark E. Reeves[1], Kimberly Payne[3], Chien Shing Chen[1], David J. Baylink[2], Guido Marcucci[4], Yi Xu[1, 2@]

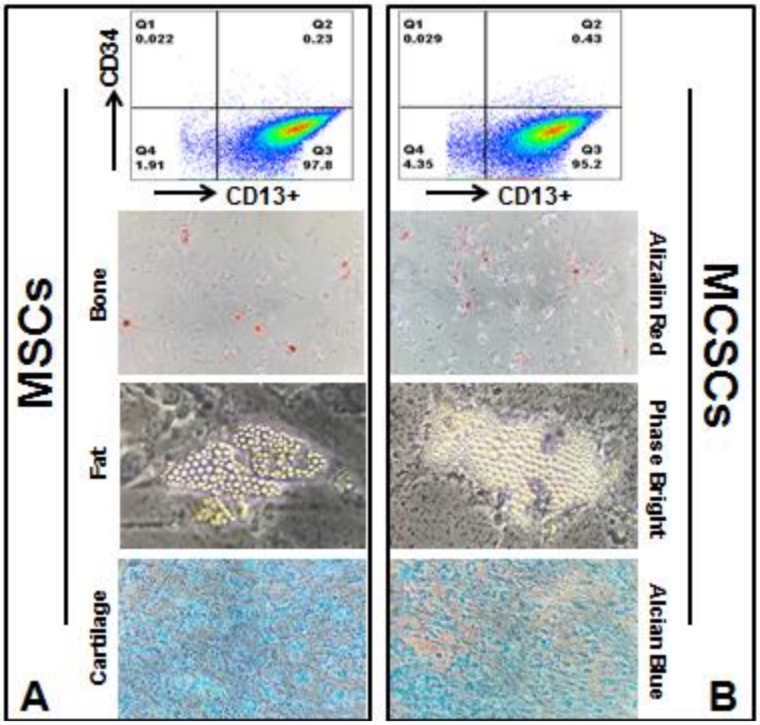

Supplementary Figure.1: There is no significant difference in differentiation capabilities of P4 MSCs and P4 MCSCs during *ex vivo* cultures.

A) MSCs Panel: FACS plot of CD34-CD13+MSCs, which differentiated into bone (Alizalin staining), fat (phase bright), and cartilage (Alcian blue staining).

B) MCSCs Panel: FACS plot of CD34-CD13+MCSCs, which differentiated into bone (Alizalin staining), fat (phase bright), and cartilage (Alcian blue staining).

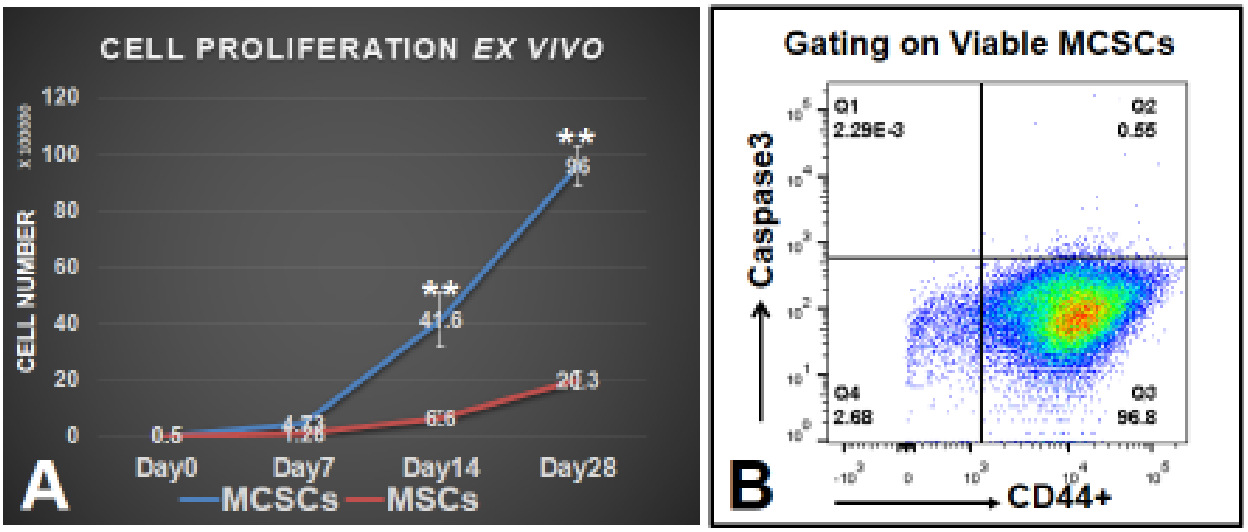

Supplementary Figure 2: Comparison of Cell Proliferation of MSCs and MCSCs.

A) The P10 MCSCs were found to proliferate much faster than P10 MSCs. ** P<0.01;

B) These rapidly proliferating MCSCs do not express cleaved Caspase3 (Cell Signaling Technology, Cat#9664S) and continue to express strong CD44+;

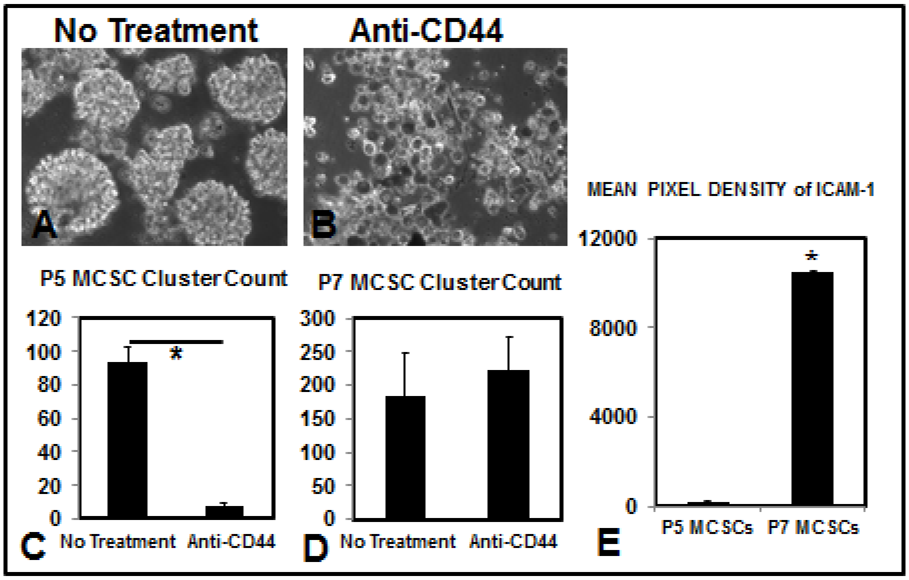

Supplementary Figure.3: Anti-CD44 monoclonal antibodies inhibited the cluster formation and proliferation of P5 MCSCs.

A) Phase bright images of floating clusters from P5 MCSCs without treatment.

B) Phase bright images of floating cells from P5 MCSCs with treatment of anti-CD44.

C) Aggregate cluster count data from P5 MCSCs treated with anti-CD44 or without treatment.

D) Aggregate cluster count data from P7 MCSCs treated with anti-CD44 or without treatment.

Where applicable, data are means ± SEM from each group and were analyzed by Student t-test.

*P<0.05; N=3.

E) Proteome comparison (mean pixel density) of ICAM-1 between supernatants from P7 MCSC and P5 MCSC cultures. *P<0.05
