# Supplementary material for: Discovery of proangiogenic CD44+mesenchymal cancer stem cells in an acute myeloid leukemia patient’s bone marrow
Source: J Hematol Oncol. 2020 Jun 3;13:63. doi: 10.1186/s13045-020-00899-x (PMC7268388; doi:10.1186/s13045-020-00899-x)
Supplement: Supplementary file 2 — Additional file 2: Materials and Methods. Discovery of proangiogenic CD44+mesenchymal cancer stem cells in an acute myeloid leukemia patient’s bone marrow [file 13045_2020_899_MOESM2_ESM.pdf]

Discovery of Proangiogenic CD44+Mesenchymal Cancer Stem Cells in an Acute Myeloid Leukemia Patient's Bone Marrow

Authors:

Huynh Cao[1*], Jeffrey Xiao[2*], Mark E. Reeves[1], Kimberly Payne[3], Chien Shing Chen[1], David J. Baylink[2], Guido Marcucci[4], Yi Xu[1, 2@]

Materials and Methods

Human samples

The AML BM sample was obtained from a donor with informed consent and approved by the Institutional Review Board at the Loma Linda University Health.

Isolation and *ex vivo* culture of MSCs and MCSCs

The AML BM sample was incubated with ACK Lysing Buffer (Catalog# A1049201, Thermo Fisher Scientific) to lyse red blood cells (RBCs). Isolation of MSCs and differentiation assays were performed according to the previous report [1]. Instead of discarding the non-adherent floating cells, we transferred the floating cells to a new 24-well plate for continuous culture for an additional 2 weeks to allow a secondary wave of cells (MCSCs) to attach to the plastic bottom of the plate. The STEMdiff™ Mesenchymal Progenitor Kit (Catalog # 05240 STEMCELLTECHNOLOGIES) was used as the culture medium. Medium change was performed every 2-3 days for both MSCs and MCSCs. Cells were passaged when reaching 80 - 90% confluence. BrdU cell proliferation assay was performed according to the manufacturer's protocol (Invitrogen™ B23151). For co-cultures, MCSCs and MSCs were mixed with the MOLM-14 AML

cell line (ATCC), genetically modified to express GFP (GFP-MOLM-14; manuscript in submission).

Cluster-forming Assay

The floating MCSC or MSC cells were re-plated at 10^5cells/mL in 24-well plates. Cell clusters were counted after 2 days, and processed for flow cytometry (FACS) assay of biomarkers (detailed protocols of FACS provided upon request).

Proteomics and Blotting Analysis

The Human XL Cytokine Array Kit (Catalog # ARY022B, R&D) was used according to manufacturer's procedures and exposed to X-ray film. Profiles of mean spot pixel density were created using a transmission-mode scanner and image analysis software.

1. Polisetti, N., et al., *Isolation, characterization and differentiation potential of rat bone marrow stromal cells.* Neurol India, 2010. **58**(2): p. 201-8.
